# Supplementary material for: Survey of the Transcription Factor Responses of Mouse Lung Alveolar Macrophages to Pneumocystis murina
Source: Pathogens. 2021 May 8;10(5):569. doi: 10.3390/pathogens10050569 (PMC8151842; doi:10.3390/pathogens10050569)
Supplement: Supplementary file 1 [file pathogens-10-00569-s001.zip › pathogens-1173519-supplementary.pdf]

Supplementary Materials:

"Survey of the Transcription Factor Response of Mouse Lung Alveolar Macrophages to *Pneumocystis murina*"

TJ Kottom, AH Limper, et al.

Table S1

Gene Name	Forward Primer	Reverse Primer
Hif1a	CCCATTCCTCATCCGTCAAATA	CCTGTGGTGACTTGTCCTTTAG
Pparg	GAAGGCTGTAAGGCTTCTTT	GTAGGCTTCGTGGATTCTCTTG
B2M	CTCGGTGACCCTGGTCTTTC	GGATTTCAATGTGAGGCGGG

Supplementary Figure S1

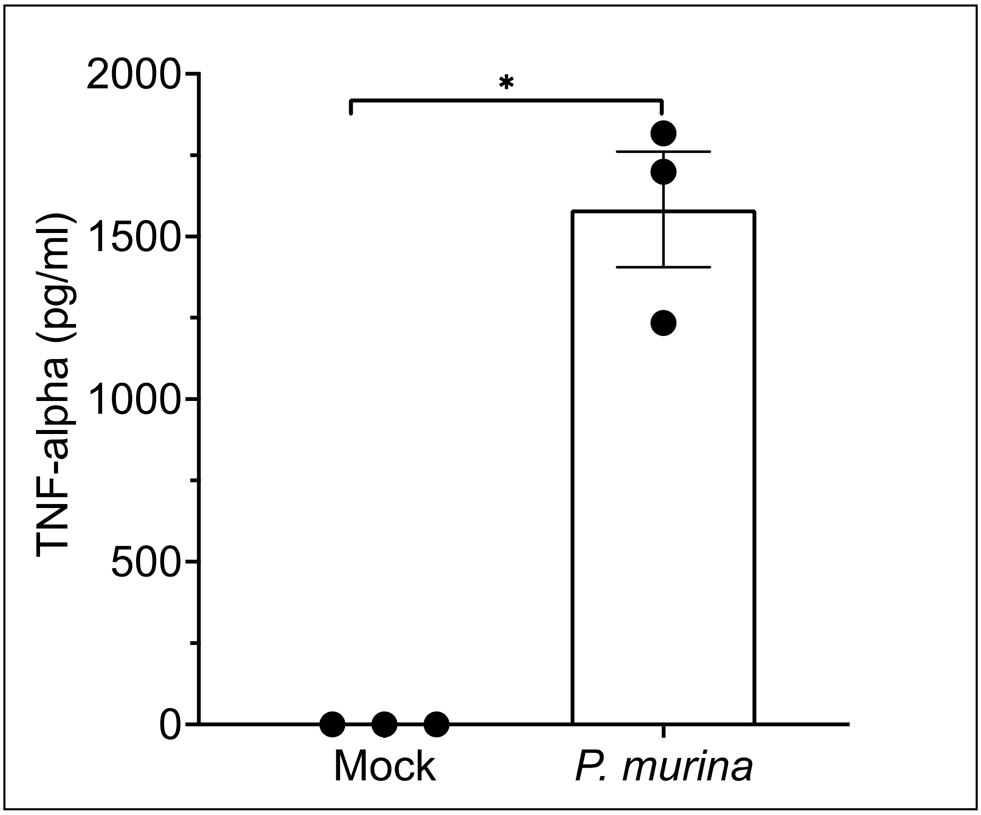

Figure S1. TNF-alpha ELISA of the supernatants of the three alveolar macrophage (AM) isolations used for the intial Qiagen RT²-PCR TF array. The bar graph represents the results from three independent experiments, with duplicate wells analyzed per condition. * $p < 0.05$.
